# Combined bisdemethoxycurcumin and potassium iodide-mediated antimicrobial photodynamic therapy

**DOI:** 10.1016/j.heliyon.2023.e17490

**Published:** 2023-06-27

**Authors:** Teerasak Damrongrungruang, Nichapat Panutyothin, Sirapakorn Kongjun, Kittapak Thanabat, Juthamat Ratha

**Affiliations:** a Division of Oral Diagnosis, Department of Oral Biomedical Science, Faculty of Dentistry, Khon Kaen University, 40002, Thailand; b Melatonin Research Program, The Research and Academic Affairs, Khon Kaen University, 40002, Thailand

**Keywords:** Bisdemethoxycurcumin, *Candida albicans*, Dental blue light, Photodynamic therapy, Potassium iodide

## Abstract

Antimicrobial photodynamic therapy is emerging as a promising way to treat infections with minimal side effects. Typically, a single photosensitizer used in photodynamic therapy is capable of generating only one type of reactive oxygen species, which may have inadequate capability to eradicate certain types of microbes, especially *Candida* species. Thus, the use of combined photosensitizers is examined as a means of achieving superior antimicrobial results. We postulate that bisdemethoxycurcumin, a type I reactive oxygen species generator, combined with potassium iodide, an antimicrobial iodide molecule, might exhibit superior antimicrobial effects compared to a single photosensitizer-mediated photodynamic therapy. The effects of bisdemethoxycurcumin + potassium iodide + dental blue light on *Candida albicans* reduction were examined. *Candida* biofilms were treated with 20, 40 or 80 μM bisdemethoxycurcumin, 100 mM potassium iodide or a combination of these species for 20 min before irradiation with a dental blue light (90 J/cm^2^). The negative and positive controls were phosphate buffer saline and nystatin at 1 : 100,000 units/ml, respectively. Candidal numbers were quantified at 0, 1, 6 and 24 h. Hydroxyl radicals were spectrophotometrically measured using 2-[6-(4′amino phynoxyl-3H-xanthen-3-on-9-yl)] benzoic acid or APF probe-mediated fluorescence intensity (Varioskan) at 490/515 nm (excitation/emission). Candidal counts and hydroxyl radical comparisons were performed using the Kruskal-Wallis test and one-way ANOVA, respectively. Correlations between candidal numbers and hydroxyl radical levels were done with a Pearson correlation test. Forty μM bisdemethoxycurcumin+100 mM KI could provide a 3.5 log_10_ CFU/ml reduction after 6 h. Bisdemethoxycurcumin alone generated OH levels that were strongly correlated with candidal reduction. In conclusion, 40 μM bisdemethoxycurcumin+100 mM KI could reduce *C. albicans* biofilm.

## Graphical abstract

**Figure:**
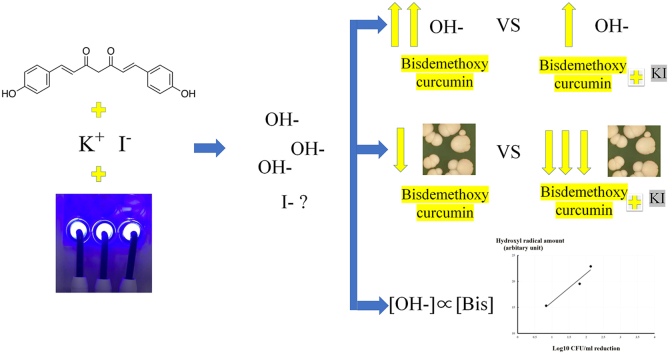

## 1 Introduction

Antimicrobial resistance issues are becoming a life-threatening problem worldwide [1]. In comparison with bacteria, some fungal species have developed stronger resistance and are very difficult to treat. Oral candidiasis, the most prevalent infection, arises from a group of species that is predominant in the oral ecology. If left untreated, malignant transformations are evident [2], especially in more susceptible hosts such as geriatric individuals or long-term users of corticosteroids. It was reported that the percentage of *Candida albican*s resistant to a standard antifungal drug (fluconazole) is approximately 30% [3] and this rate substantially increases in immunocompromised individuals.

Antimicrobial photodynamic therapy (aPDT) [4], a novel treatment, is emerging as an effective means to counteract oral infections, including oral candidiasis [5]. Based primarily on the specificity of a photosensitizer that binds to oral microorganisms, but not human host cells, this approach could lead to a targeted therapy with neither adverse reactions nor drug resistance issues. To the best of our knowledge, resistance to aPDT by *C. albicans* has never been reported.

PDT reactions rely primarily on light photon absorption by a photosensitizer (PS), which cascades multiple reactions. This in turn stimulates both type I and type II reactive oxygen species (ROS) formation resulting in hydroxyl radical (OH^−^) and singlet oxygen (^1^O_2_) formation, respectively, based upon whether electrons or energy are transferred [6]. Basically, each photosensitizer, in a specific chemical form and pH condition, can preferentially generate a reactive oxygen species product *via* only one of these reaction types. For instance, methylene blue in a monomeric form can generate singlet oxygen (a type II product). However, in a dimeric form within a strongly acidic environment, the type II reaction is shifted to a type I reaction, practically ending singlet oxygen generation [7]. The typical examples are curcumin from Turmeric, which can generate hydroxyl radicals, and erythrosine, the most used oral microbial biofilm disclosing agent [5], which is able to generate singlet oxygen [8] as its main product. Curcuminoids have almost ideal photosensitizer properties because of their photokilling potential and low dark toxicity [9] as well as preferential uptake by cancer cells/microbial cells compared to normal human cells [10]. The therapeutic efficacy derived from a single PS with monotype reaction products might not be effective to eradicate oral candidal infections. Recently, a combined PS strategy was introduced to improve antifungal therapeutic efficacy [5,11]. An example of an effective strategy is to combine photosensitizers with both type I and type II reactions to simultaneously maximize ROS. It is generally accepted that curcumin derivatives possess antimicrobial activities, especially from bisdemethoxycurcumin. This is presumably due to its superior adherence to the peptidoglycan on candidal cells [12]. A recent report demonstrated that a combination of 20 μM bisdemethoxycurcumin, 110 μM erythrosine, and 10% (w/w) nano titanium dioxide irradiated at 67 J/cm^2^ at a wavelength of 420–480 nm using a dental light emitting diode (LED) significantly reduced a *C. albicans* biofilm by approximately 1.1 log_10_ CFU/ml [5]. This reduction, however, is still far from a 3 log_10_ CFU/ml reduction, which is the standard level proposed by the American Association for Microbiology [13].

It was previously reported that a combination of potassium iodide (KI) with methylene blue or Rose Bengal could potentiate candidal reduction [14]. KI is a non-toxic salt with antimicrobial properties. This substance is generally used for the treatment of thyroid disease. In photodynamic therapy, upon photon absorption, KI can generate singlet oxygen as well as peroxyl iodide [14]. KI could enhance a planktonic candida cell reduction of 6 log_10_ colony forming units (CFU)/ml [15], but for a candidal biofilm, addition of 100 mM KI with methylene blue with irradiation energy of 10–60 J (energy density of 3.18–19.1 J/cm^2^) could reduce candida numbers by up to 2 log_10_ CFU/ml [16]. Bisdemethoxycurcumin is a hydrophobic molecule. A previous study utilized dimethyl sulfoxide (DMSO) [17] and acetone [18] as solvents, but due to the toxicity of DMSO in human cells, we decided to use ethanol as a solvent instead. It would, therefore, be of great interest to investigate the photodynamic potential of bisdemethoxycurcumin combined with KI to exert clinically acceptable antifungal activity.

Dental blue LEDs, with a wavelength range from 420 to 480 nm, are widely used in all dental clinics. Kazantzis et al. found that the maximum absorption wavelength of bisdemethoxycurcumin is 426 nm [17], which is within the spectrum of a dental blue LED. Also, they found that using blue light, which has a high molar extinction coefficient for bisdemethoxycurcumin, could stimulate generation of a higher amount of ROS. This is one of the desired characteristics of PS [17].

The current study presents the results of candidal (American Type Culture Collection; ATCC 10231) biofilm reduction at 0–24 h using various concentrations of bisdemethoxycurcumin with KI in the presence of a continuously operated blue dental LED with an energy density 95 J/cm^2^. We also correlated the candidal reducing effect with hydroxy radical generation.

## 2 Materials and methods

### 2.1 Photosensitizers

Bisdemethoxycurcumin was purchased from MedChem Express (Suite Q, NJ, USA) and potassium iodide was obtained from Sigma (Sigma-Aldrich, NJ, USA).

Bisdemethoxycurcumin was dissolved in absolute ethanol (final concentration, <1% ethanol) and heated at 70 °C for 5 min to prepare a stock solution. Then, deionized water was added to make a 160 μM solution. Potassium iodide was dissolved in deionized water to make a 400 mM stock solution. The treatment groups were as follows.

#### 2.1.1 Experimental groups

Bisdemethoxycurcumin 20 μM + blue LED.

Bisdemethoxycurcumin 40 μM + blue LED.

Bsisdemethoxycurcumin 80 μM + blue LED.

Potassium iodide 100 mM + blue LED.

Bisdemethoxycurcumin 20 μM + Potassium iodide 100 mM + blue LED.

Bisdemethoxycurcumin 40 μM + Potassium iodide 100 mM + blue LED.

Bisdemethoxycurcumin 80 μM + Potassium iodide 100 mM + blue LED.

#### 2.1.2 Control groups

Positive control – Nystatin 100,000 U/ml.

#### 2.1.3 Negative control – phosphate buffer saline

All solutions were freshly prepared prior to use and kept in containers wrapped with aluminum foil to avoid premature reaction with light.

### 2.2 Photodynamic parameters

**Fig. 1:**
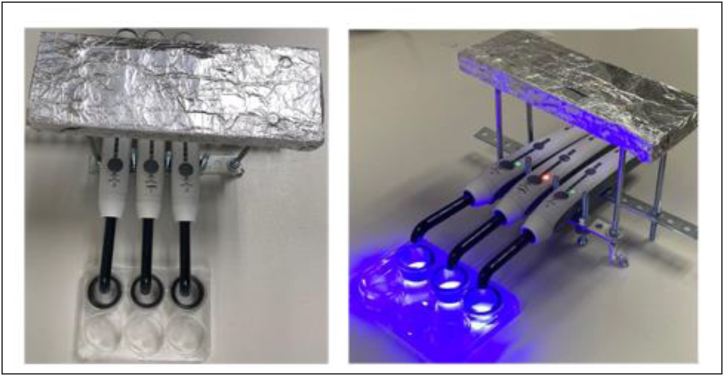
Light source mounting apparatus equipped with a black ring. Top (left) and lateral views (right) of this apparatus allow three simultaneous irradiation treatments in a 6-well plate with a black ring to prevent scatter of radiation to adjacent wells. The vertical distance from the tip of the light source to the bottom of the well can be adjusted.

Blue dental resin composite light curing units (Elipar™ DeepCure-L Curing Light, 3M™, Singapore) were mounted on a lab-constructed apparatus (Fig. 1) with a light source to bottom-of-well distance of 25 mm. The power density was 950 mW/cm^2^ and irradiation time was fixed at 95 s, thus the average energy density was 90 J/cm^2^.

### 2.3 Hydroxyl radical measurement using a fluorescence method

The probe used for hydroxyl radical measurement was 2-[6-(4′amino phynoxyl-3H-xanthen-3-on-9-yl)] benzoic acid or APF (Thermofisher, Waltham, MA, USA). APF and dimethylformamide (DMF) were mixed in a 1 : 4 vol ratio to make a 1 mM solution of APF in DMF. Next, 10 μL of the APF probe were then mixed with 100 μl of 10× (10 times higher concentration of each of the above samples (in section 2.1). Finally, 890 μl of deionized water was added, yielding an APF probe final concentration of 10 μM). Immediately, each group was irradiated according to the previously presented light parameter and fluorescent intensity (FI) protocols measured at emission and excitation wavelengths 490 and 515 nm using a spectrophotometer (Varioscan®, Thermofisher Scientific, Waltham, MA, USA). Measurements were performed every min from 0 to 5 min and the average FI was calculated. Ten μM of a standard curcumin solution and probe served only as positive and negative controls, respectively.

### 2.4 C. albicans biofilm culture

*Candida albicans* strain ATCC 10231 was cultured in Sabouraud dextrose broth (SDB) until it achieved an optical density of 0.38 at a 520 nm wavelength, which corresponds to a candidal cell count of 1 × 10^7^ cells/ml. Subsequently, 1 ml of this candidal cell culture in SDB was plated onto a pre-weighed glass slide, placed into a black six-well plate and incubated at 37 °C on a 75round/min (rpm) shaking incubator for 90 min. Phosphate buffer saline washing was performed twice and the cells were then treated with 2 ml of a biofilm-enriching solution (yeast nitrogen base (1×) and 50 mM glucose) for 48 h in an incubator at 37 °C with a relative humidity ≥90%. Mature biofilm weight was recorded.

### 2.5 Photodynamic therapy on the candida biofilm

Mature candidal biofilm was washed twice using 2 ml of PBS before being treated with 2 ml of samples of each PS in SDB. The biofilm samples were kept under a relatively dark condition for 20 min prior to continuous irradiation with a blue dental LED for 95 s. A light power meter (PM160T-HP Thorlabs, Newton, NJ, USA.) was used to check the light intensity daily before experimentation. The temperature of all processes was measured and a subtle temperature increase of <4 °C was observed. Positive and negative controls were 1 : 100,000 U/ml of nystatin and SDB, respectively.

### 2.6 Candidal colony counting

At 0, 1, 6, and 24 h post-irradiation, the plates were ultrasonicated in distilled water for 15 min. Then, the resulting solutions were centrifuged at 8000 rpm for 5 min. Finally, the supernatants were discarded, and the precipitates were subjected to 10-fold serial dilutions resulting in undiluted solutions and 10^−1^, 10^−2^, and 10^−3^ dilutions. Three 10-μl drops of each concentration were plated onto sterilized Sabouraud dextrose agar and subsequently cultured in an incubator at 37 °C with humidity >90% for 48 h Colony counting with logarithmic transformation was conducted.

### 2.7 Statistical analysis

Candida numbers were expressed as median and interquartile range of log_10_ colony forming units/ml that were normalized by biofilm weight and compared at 0, 1, 6, and 24 h using a Kruskal-Wallis test with further *post hoc* testing.

Relative hydroxyl amounts were analyzed by comparison of mean and standard deviation at each time point. All comparisons were determined as statistically significant differences at p < 0.05.

The relationship between hydroxyl radical amounts and candidal cells counts was determined using a Pearson correlation test.

## 3 Results

### 3.1 Hydroxyl radical quantification using a fluorescence method

**Fig. 2:**
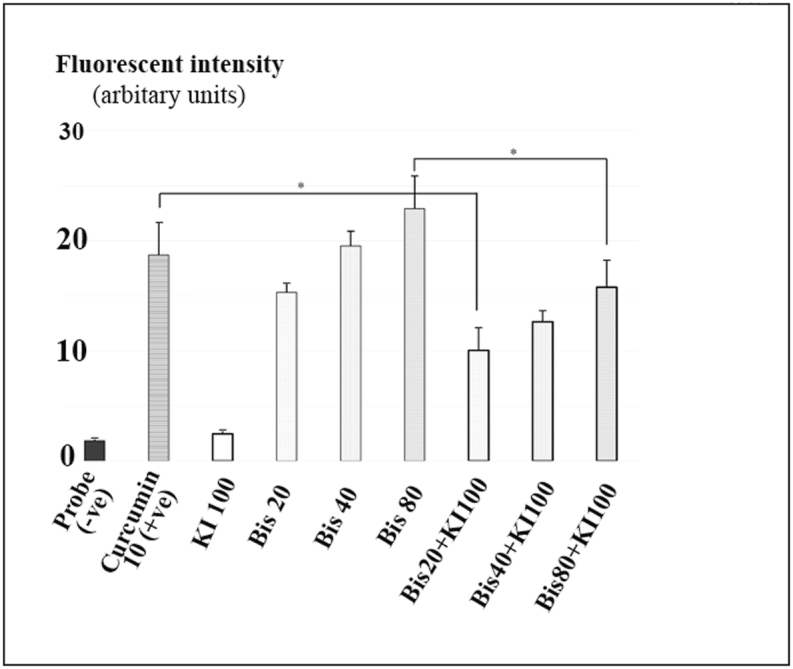
Comparison of average hydroxyl radical quantities using a fluorescence method (an *in vitro* assay). Hydroxyl radicals were immediately measured in a dim environment in arbitrary units of fluorescence intensity (performed every minute for 5 min) at excitation and emission wavelengths of 490 and 515 nm, respectively using a Varioskan spectrophotometer. The negative and positive controls were 10 μM of an APF probe in dimethylformamide and 10 μM curcumin in ethanol, respectively (n = 9). * indicates a statistically significant difference at p < 0.05.

Fig. 2 demonstrates relative hydroxyl radical quantification using an APF probe. A higher a fluorescence intensity from the APF probe that is substituted its aminobenzene ring by hydroxyl radical indicates a greater amount of hydroxyl radicals present. It clearly shows that this ROS species increased in a dose-dependent manner. The highest amount of hydroxyl was derived from 80 μM of bisdemethoxycurcumin (22.91 ± 2.98 arbitrary units). Both the 40 and 80 μM of bisdemethoxycurcumin treatments generated hydroxyl radicals equal to 10 μM curcumin (positive control) while KI could not generate a detectable level of hydroxyl radicals.

Notably, addition of 100 mM of KI to bisdemethoxycurcumin resulted in reduced hydroxyl radical levels to approximately 30–48% of the amount produced by bisdemethoxycurcumin alone at the same concentration. Eighty μM bisdemethoxycurcumin+100 mM KI generated a lower and statistically significant amount of hydroxyl radicals compared with 80 μM bisdemethoxycurcumin alone, p = 0.016. Interestingly, when compared with the positive control (10 μM of curcumin), 20 μM bisdemethoxycurcumin+100 mM KI presented a significantly reduced level of hydroxyl radicals (p = 0.01).

### 3.2 Candida albicans reduction by photodynamic therapy using bisdemethoxycurcumin, KI, and blue LED

Most of test groups showed reduced *Candida* biofilms compared with pretreatment or baseline conditions (p < 0.05, all). The maximum reduction of the *Candida* biofilm was achieved using 40 μM of bisdemethoxycurcumin+100 mM KI for 1 h. This treatment showed a candidal reduction of 3.5 log_10_ CFU/ml, while 20 μM + 100 mM KI reduced *Candida* biofilm by only 0.52 log_10_ CFU/ml after 1 h. The groups that were able to dramatically reduce candidal biofilms to a degree comparable to nystatin were 40 μM of bisdemethoxycurcumin+100 mM KI at all time points, 80 μM of bisdemethoxycurcumin both with and without 100 mM KI after 24 h, along with 20 and 40 μM bisdemethoxycurcumin for 24 h. On the contrary, the groups that have almost no ability to suppress *C. albicans* are 100 mM KI at all time points, 20 μM of bisdemethoxycurcumin at 0 and 1 h and 40 μM of bisdemethoxycurcumin treatment at 0 h as well as a compound treatment of 20 μM bisdemethoxucurcumin+100 mM KI at 0 and 1 h.

**Fig. 3:**
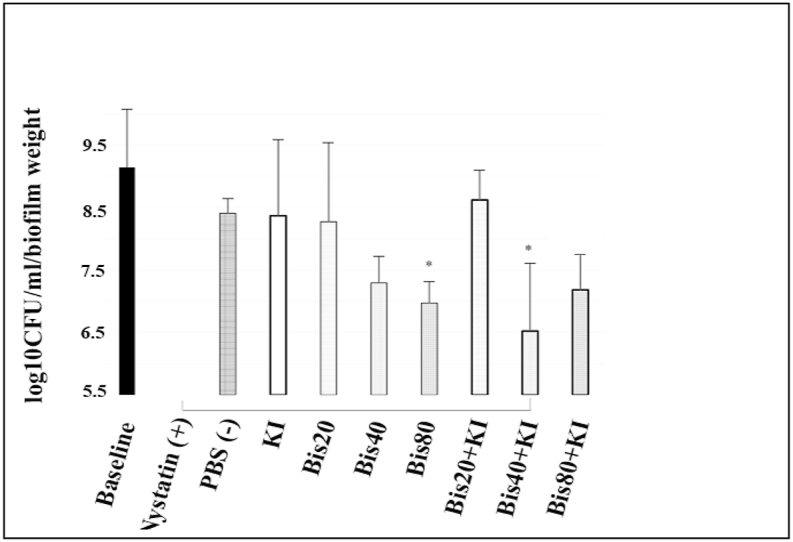
Comparison of *C. albicans* (median ± IQR) in log_10_ CFU/ml/biofilm weight at 0 h post-PDT (blue LED, 950 mW/cm^2^, 90 J/cm^2^). A drop plate assay was used. The positive and negative controls are 1 : 100,000 U/ml of nystatin and phosphate buffer saline, respectively. * indicates a statistically significant difference from PBS (negative control) at p < 0.05. A solid line under the bar between nystatin and other groups indicates a non-significant difference from nystatin (n = 5).

Fig. 3 shows the candidal counts immediately after photodynamic therapy. Twenty μM of bisdemethoxycurcumin, 100 mM of KI, and 20 μM bisdemethoxycurcumin+100 mM of KI did not reduce candidal counts when compared with the negative control (PBS) group. While 80 μM bisdemethoxycurcumin, as well as 40 μM bisdemethoxycurcumin+100 mM KI showed statistically significant results, reducing candida by about 1.44 and 1.89 log_10_ CFU/ml (p = 0.016 and 0.003), respectively, compared to the negative control (PBS). Notably, 40 μM bisdemethoxycurcumin+100 mM of KI could reduce *Candida* counts to levels equal to the positive control (1 : 100,000 Unit nystatin).

**Fig. 4:**
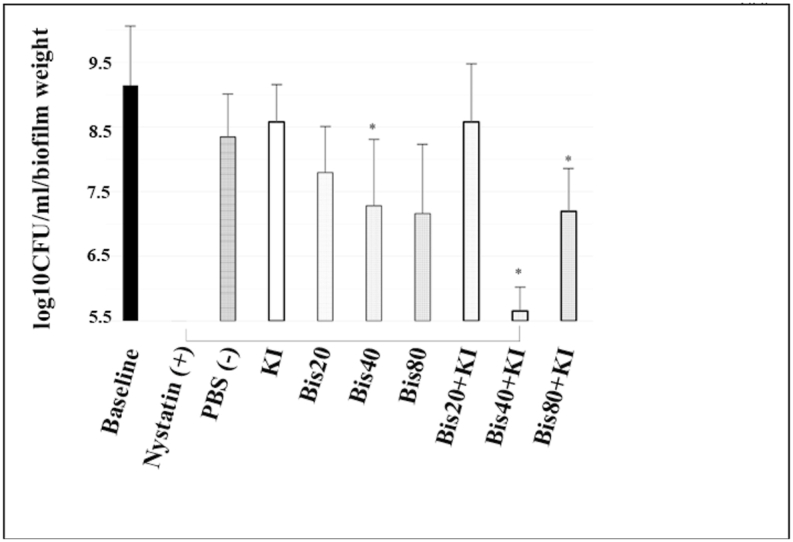
Comparison of *C. albicans* (median ± IQR) in log_10_ CFU/ml/biofilm weight after 1 h post-PDT (blue LED, 950 mW/cm^2^, 90 J/cm^2^). A drop plate assay was used. Positive and negative controls are 1 : 100,000 U/ml of nystatin and phosphate buffer saline, respectively. * indicates a statistically significant difference from PBS (negative control) at p < 0.05. A solid line under the bar between nystatin and other groups indicates a non-significant difference from nystatin (n = 5).

At 1 h post-PDT, as shown in Fig. 4, the same trends were observed as at 0 h in the single substance group, in which higher concentrations of bisdemethoxycurcumin showed a greater reduction of *Candida*. Notably, 40 μM of bisdemethoxycurcumin could reduce *Candida* levels by 1.07 log_10_ CFU/ml which is a significant difference from the baseline (p = 0.036). A combination compounds comprising 40 μM bisdemethoxycurcumin+100 mM KI and 80 μM bisdemethoxycurcumin+100 mM KI could significantly reduce candidal numbers by 2.69 and 1.15 log_10_ CFU/ml (p < 0.001 and p = 0.027) compared with PBS. The former group especially showed a reduction capability that is comparable to the effect of nystatin. KI at 100 mM as well as 20 μM bisdemethoxycurcumin did not affect candidal reduction.

**Fig. 5:**
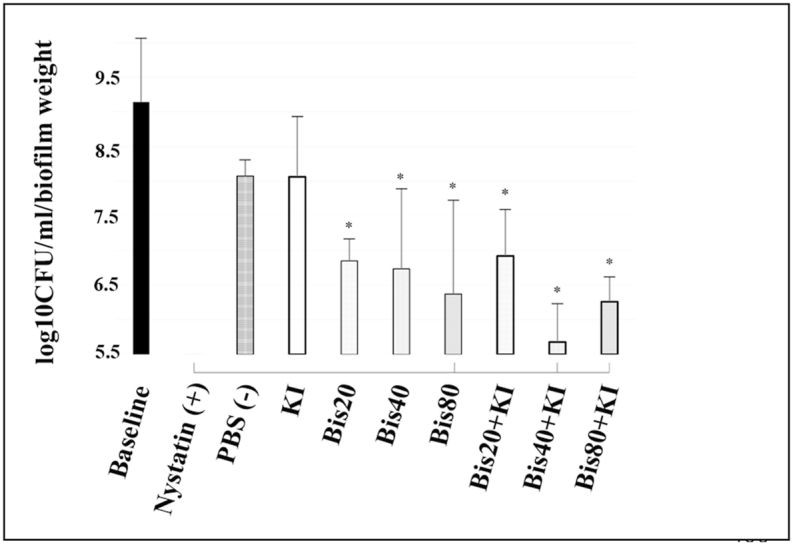
Comparison of *C. albicans* (median ± IQR) in log_10_ CFU/ml/biofilm weight at 6 h post-PDT (blue LED, 950 mW/cm^2^, 90 J/cm^2^). A drop plate assay was used. Positive and negative controls are 1 : 100,000 U/ml of nystatin and phosphate buffer saline, respectively. * indicates a statistically significant difference from PBS (negative control) at p < 0.05. The solid line under the bars between nystatin and other groups indicates a non-significant difference from nystatin (n = 5).

After 6 h of PDT (Fig. 5), both single and combination PSs showed higher levels of candidal reduction that were significantly different from the baseline and the negative control. For a single compound, a dose-dependent reduction of *Candida* was observed. The maximal reduction was still for a treatment of 40 μM bisdemethoxycurcumin+100 mM KI that reduced *Candida* by 2.4 log_10_ CFU/ml (p < 0.01, all) compared to the negative control. Notably, higher concentrations (80 μM of bisdemethoxycurcumin with or without 100 mM KI) could reduce *Candida* by 1.7 and 1.81 log_10_ CFU/ml, respectively (p < 0.001, both) compared with PBS (negative control). All three groups had a candidal reducing capability comparable to that of the nystatin standard. Again, 100 mM of KI also did not reduce the *Candida* count at this time point.

**Fig. 6:**
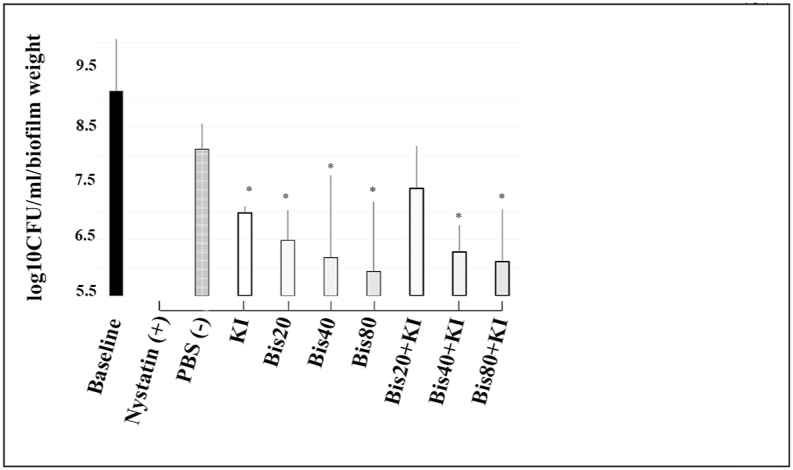
Comparison of *C. albicans* (median ± IQR) in log_10_ CFU/ml/biofilm weight at 24 h post-PDT (blue LED, 950 mW/cm^2^, 90 J/cm^2^). A drop plate assay was used. Positive and negative controls are 1 : 100,000 U/ml of nystatin and phosphate buffer saline, respectively. * indicates a statistically significant difference from PBS (negative control) at p < 0.05. The solid line under the bars between nystatin and other groups indicates a non-significant difference from nystatin (n = 5).

At 24 h, all interventions, except 20 μM bisdemethoxycurcumin+100 mM KI, dramatically reduced *Candida* levels (Fig. 6). The single PS group exerted more candidal reduction at 6 h. Notably, 100 mM KI alone could also significantly reduce candidal numbers by 1.13 log_10_ CFU/ml compared to PBS (p = 0.015). Forty and 80 μM of bisdemethoxycurcumin with and without 100 mM KI could reduce *Candida* growth by 1.83–2.18 log_10_ CFU/ml compared to the negative control, which achieved a statistically significant difference (p < 0.01, all) compared to PBS (negative control). Also, these four groups presented candidal reduction capability equal to that of nystatin. Interestingly, candidal reduction by 40 μM bisdemethoxycurcumin+100 mM KI was less than at 6 h (2.4 compared with 1.83 log_10_ CFU/ml).

**Fig. 7:**
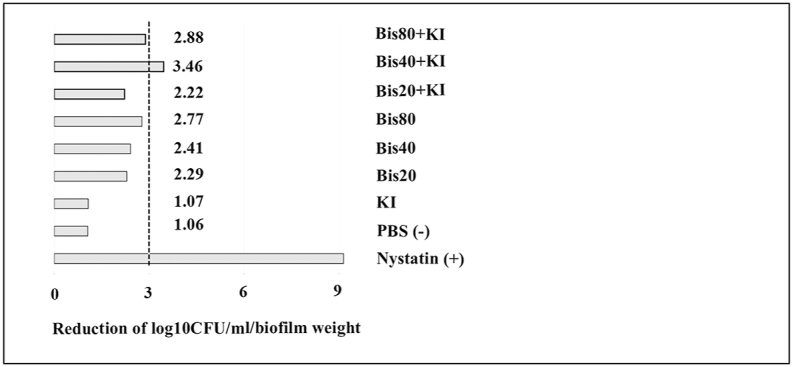
Comparison of *C. albicans* reduction (median ± IQR) in log_10_ CFU/ml/biofilm weight at 6 h post-PDT (blue LED, 950 mW/cm^2^, 90 J/cm^2^) compared with the baseline. A drop plate assay was used. Positive and negative controls are 1 : 100,000 U/ml of nystatin and phosphate buffer saline, respectively, (n = 5). The dotted line represents a 3 log_10_ CFUL/ml reduction.

The reduction of *C. albicans* compared to the baseline after 6 h is shown in Fig. 7. It ranged from 1.06 to 3.46 log_10_ CFU/ml/biofilm weight. Maximal reduction was observed in the group with 40 μM bisdemethoxycurcumin+100 mM of KI. Bisdemethoxycurcumin with and without KI could reduce candidal numbers to almost the same degree, except for the 40 μM group, where addition of KI dramatically decreased the biofilm.

### 3.3 Correlation between hydroxyl radical level and Candida albicans reduction

**Fig. 8:**
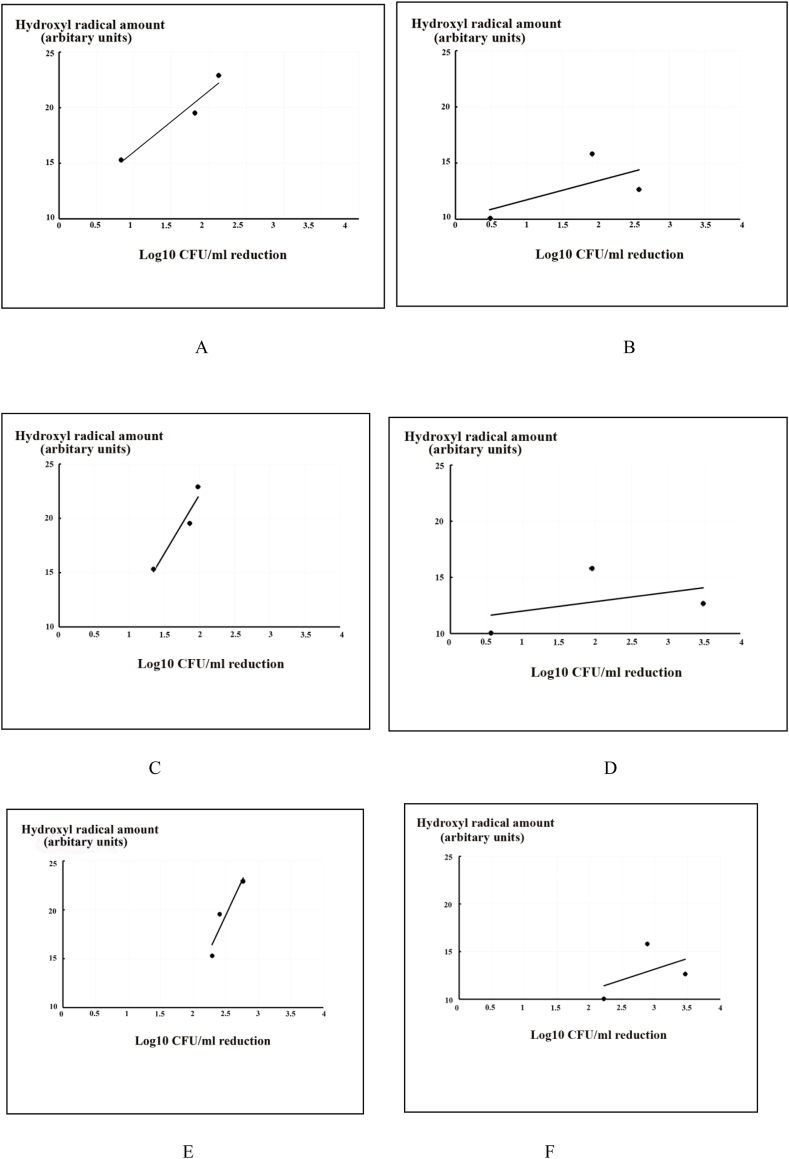

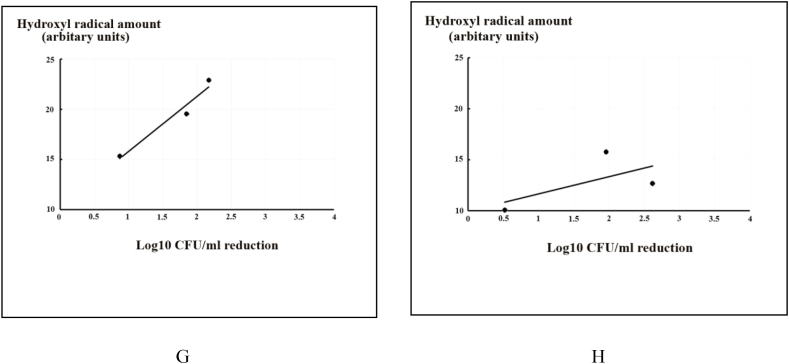
Correlation between hydroxyl radical production and candidal counts as a log_10_ CFU/ml reduction at 0 h (A,B), 1 h (C,D), 6 h (E,F), and 24 h (G,H) after PDT reaction using blue LED 420–480 nm at an energy density of 90 J/cm^2^. A,C,E,G: Bisdemethoxycurcumin only, and B,D,F,H: Bisdemethoxycurcumin + KI (n = 5).

Fig. 8A demonstrates the correlation between the effect of a single substance and *C. albicans* reduction, while Fig. 8B demonstrated the effect of combined substances on *C. albicans* and hydroxyl radical levels. For bisdemethoxycurcumin alone, it can be clearly seen that a greater concentration of bisdemethoxycurcumin in the system is correlated with higher hydroxyl radical production at a high correlation coefficient (r = 0.98). Additionally, a combination of bisdemethoxycurcumin and KI demonstrated a moderate degree of correlation with hydroxyl radical production (r = 0.63).

After 1 h, correlations between the reduction of *C. albicans* and hydroxyl radical production from single and combined substances are shown in Fig. 8C and D, respectively. The correlation coefficient in the single substance group was r = 0.96, or strongly correlated, which is similar to that at 0 h. However, that of the combination groups at the same time points was r = 0.43, representing a decreased, but still moderate correlation.

After 6 h, the correlation of the reduction of *C. albicans* and hydroxyl radical produced from single and combined substances still shows the same trend as those at 0 and 1 h, with correlations of 0.94 and 0.49, strong and moderate correlations, respectively (Fig. 8E and F).

After 24 h, the correlation of the reduction of *C. albicans* and hydroxyl radical produced from single and combined substances showed the same trend as after 0 and 1 h, with correlation coefficients of r = 0.98 and r = 0.63, which are strong and moderate, respectively (Fig. 8G and H). Notably, the correlation of the combined group was moderate, but nearly a strong correlation.

## 4 Discussion

The present study aims to investigate the effectiveness of using bisdemethoxycurcumin and potassium iodide together for in a single photodynamic therapy. Bisdemethoxycurcumin generates type I photodynamic therapy reactive oxygen species when exposed to dental blue LED [5], while potassium iodide may generate iodide derivatives. These iodides react with the hydroxyl radicals from bisdemethoxycurcumin and might give rise to short-lived reactive iodide species that, in turn, promote a small microbial inhibitory effect [14]. Additionally, the negatively charged nature of the *Candida* surface [19] can attract the positive charges of potassium in KI, leading to binding and inactivation of *Candida*. Thus, in combination, they may exert a superior antifungal effect. The current study successfully demonstrates this synergistic effect of a photosensitizer with KI as a potentiator of antimicrobial PDT on reduction of a *C. albicans* biofilm *in vitro* as a result of photodynamic therapy.

In terms of candidal biofilm reduction, 40 μM bisdemethoxycurcumin in combination with 100 mM KI was a treatment that yielded a maximum effect of an approximately 3.5 log_10_ CFU/ml reduction. Based on our findings, more bisdemethoxycurcumin in PDT generally produced higher hydroxyl radical levels. The inhibitory capability of 40 μM bisdemethoxycurcumin, in combination with 100 mM KI, is likely superior to that of 80 μM bisdemethoxycurcumin with the same quantity of KI. This is possibly due in part to the test parameters, themselves. There might be a nonequilibrium between the applied light energy and amount of photosensitizer. Some bisdemethoxycurcumin remained unreacted after the PDT reaction was completed. Natural bisdemethoxycurcumin itself can act as an ROS scavenger [12], which has been linked to lower levels of ROS generated and hence lower candidal reduction.

In a previous study by Kanpittaya et al., 20 μM bisdemethoxycurcumin was used in combination with 110 μM of erythrosine. They demonstrated only a 1.12 log_10_ CFU/ml candidal biofilm reduction after 24 h [5]. The present study also used the same concentration, 20 μM bisdemethoxycurcumin, but added 100 mM KI. It was found that after 24 h, this combination yielded an approximately 0.61 log_10_ CFU/ml higher reduction. However, when compared with reduction at 6 h post-PDT, our 20 μM bisdemethoxycurcumin+100 mM KI treatment showed a 1.1 log_10_ CFU/ml higher reduction of *Candida*. This might be explained in part that KI in millimolar concentrations can generate a large amount of iodide derivatives [14] that negatively impact *Candida* cells. Also, the irradiation time in a previous study was relatively short (only 21 s), while our study used 95 s. Thus, the photodynamic reaction in the present study might be relatively completed. Another interesting point is that the energy density in their study was lower than in the present study (72 J/cm^2^ *vs.* 95 J/cm^2^), which might be inadequate to generate sufficient ROS for reduction of *C. albicans*.

Nystatin is a standard antifungal agent used in clinical practice. The present study demonstrated that this drug could reduce *Candida* levels by more than 6 log_10_ CFU/ml in as little as 1 h. Our combined photosensitizers and blue LED at the same time point, however, still have lower potency in terms of candidal biofilm reduction. This can be explained since the light source used in the present study cannot be well-absorbed by KI, hence the production of iodide derivatives might be at low level. The maximum absorption wavelength of KI is in the UVA range [20]. A previous study demonstrated that 100 mM of KI with nano-titanium dioxide could decrease *Candida* by 4 log_10_ CFU/ml in planktonic form compared with treatments with and without 100 mM KI [21]. Even though this was quite a profound effect, planktonic cells are highly susceptible to antifungal agents, while biofilms are 30–2000 times more resistant [22]. Thus, the present study demonstrated a superior *anti*-candidal effect to the above-mentioned study [21]. Moreover, UV light is harmful to humans. Our study used a dental LED, which is safe and is an already available light source in all dental clinics. Nevertheless, addition of more photosensitizers, especially those that generate type II ROS, is worth further investigation to achieve a candidal reduction potency equivalent to nystatin.

A previous study compared pre-irradiation times. It found that the remaining candidal cells in planktonic form were reduced from 2.10 ± 0.17 log_10_ CFU/ml to 0.60 ± 0.79 log_10_ CFU/ml when the pre-irradiation time was increased from 5 to 20 min [23]. A *Candida* biofilm is a complex structure. Bisdemethoxycurcumin needs time to penetrate a biofilm and its cells, which is why we selected a pre-irradiation time of 20 min in our study. Although this period was relatively long, it allowed the photosensitizer accumulate at a maximal level to receive light energy. Low power light irradiation immediately after the application of photosensitizers that gradually increases might decrease clinical chair time as well as enhance PDT efficacy. This is based on metronomic photodynamic therapy (mPDT) [24].

For reactive oxygen species, a previous study used Photofrin, a hematoporphyrin-based substance that is known to generate singlet oxygen and exerts antimicrobial effects, to inhibit *Candida*. In their study, KI was also added [25]. Singlet oxygen subsequently stimulated potassium iodide generation of peroxyiodide and affected the reduction of *Candida* [25]. Photofrin, however, has a large molecular structure that has a relatively long drug-light interval of at least 30 min [26] to ensure complete absorption into candidal cells. The oxygen quantum yield of Photofrin [27] was as low as 0.11, while the hydroxyl radical quantum yield (in ethanol) of bisdemethoxycurcumin is about 0.143 [28]. Even though direct comparison cannot be performed, this presumably demonstrates that bisdemethoxycurcumin with KI may produce a higher amount of ROS products and hence, collectively exert a superior inhibitory effect on *Candida*.

It was clearly demonstrated that longer post-photodynamic therapy resulted in a higher reduction in *Candida* counts. Previous studies showed that hydroxyl radicals were detected within several minutes [29], but hydroxyl radicals can react with other biomolecules [30] to exert effects over a longer period than its lifetime. It is generally established that two hydroxyl radicals can form hydrogen peroxide, which is more stable and lasts for more than 24 h [31]. Additionally, hydroxyl radicals can gradually be converted into singlet oxygen and exert an antimicrobial effect *via* the Fenton reaction. It is generally accepted that higher hydroxyl radical concentrations lead to superior antimicrobial effects. Our results are consistent with this fact, because among the combination groups, the treatment that generated the highest amount of hydroxyl radicals was the one that could most reduce *C. albicans*. However, when compared with a single PS that produced a higher amount of hydroxyl radicals, the combination groups with higher concentrations of bisdemethoxycurcumin at 1–6 h exhibited superior candidal inhibition. It is conceivable that, as mentioned earlier, some generated hydroxyl radicals and oxygen in the environment might react with KI to produce I_2_ and a hydroperoxyl radical. The former molecule is generally accepted as bactericidal and fungicidal *via* complement system-mediated cell lysis [32]. The latter molecule can induce mitochondria deficiency in yeast [33]. This leads only to a moderate correlation between the hydroxyl radical and candidal number in the combination treatments, while a strong correlation was found for bisdemethoxycurumin alone. Measuring iodine and iodide derivatives as well as hydroperoxyl radicals is crucial to data to collect. Not only does ROS plays a role in oxidative stress, but reactive nitrogen species (RNS) also orchestrate this process. Determination of the RNS level might explain this reaction.

For human cell toxicity, it was reported that using 80 J/cm^2^ in a sustained release curcumin nanoemulsion had no effect on MCF-7 breast cancer cells [34]. Also, it was reported that 70 μM of either curcumin or bisdemethoxycurcumin was nontoxic to osteoblast cells [35]. The present study utilized the same energy range as previously studies, thus our regimen should also not present toxicity to normal human oral cells. We are now, however, confirming the toxicity of our regimen with oral keratinocytes. Since the effect in the present study remains for up to 6 h, development of a sustained release system *e.g.*, a nanoemulsion, is worth study.

A strength of the present study was the use of both bisdemethoxycurcumin and KI, which might have good adhesion to the candidal cell surfaces, leading to a prolonged anticandidal effect. It also uses an already available light source that is easy to apply in a clinical setting. However, the present study also has some limitations. The measurements of hydroxyl radical were conducted *in vitro* and might not directly correlate with the amount of hydroxyl radical produced *in vivo* (in normal candidal cells). Additionally, the molecular interactions between bisdemethoxycurcumin and KI have not been examined. Therefore, it would be beneficial to use molecular docking to clearly explain the results before applying this method in clinical practice.

## 5 Conclusion

In conclusion, 40 μM of bisdemethoxycurcumin in combination with 100 mM potassium iodide with 90 J/cm^2^ dental blue light could effectively reduce a *C. albicans* biofilm after 6 h with an efficacy equivalent to nystatin.

## CRediT author contribution statement

Teerasak Damrongrungruang: Conceptualization, Methodology, Supervision, Project administration, Funding acquisition, Resources, Validation, Writing- Reviewing and Editing Nichapat Panutyotin: Data curation, Formal analysis, Investigation, Writing- Original draft preparation, Visualization. Sarapakorn Kongjun: Formal analysis, Visualization, Investigation、Writing-Original draft preparation Kittapak Thanabat: Investigation. Validation, Writing – original draft Juthamat Ratha: Methodology, Data curation, Software, Validation.

## Funding

This research was financially supported by the Fundamental Fund of 10.13039/501100004071Khon Kaen University, the National Science, Research and Innovation Fund (NSRF), and the 30th Anniversary Fund of Faculty of Dentistry, 10.13039/501100004071Khon Kaen University [Grant No. DT6503]. Khon Kaen, Thailand.

## Data availability statement

Data will be made available on request.

## Graphic abstract

Combination of bisdemethoxycurcumin and KI with dental blue light in photodynamic therapy showed enhanced hydroxyl radical production. This combined with photosensitizers effectively reduced C. albicans biofilms to a greater degree than single photosensitizers and is almost equivalent to nystatin. A strong correlation between hydroxyl radicals and candidal biofilm reduction was seen only in the single bisdemethoxycurcumin group.

## Declaration of competing interest

The authors declare that they have no known competing financial interests or personal relationships that could have appeared to influence the work reported in this paper.
